# Supplementary material for: Are carbon emissions trading and green financial instruments synergistic? -Comprehensive quantitative research based on content analysis
Source: PLoS One. 2024 Mar 7;19(3):e0298601. doi: 10.1371/journal.pone.0298601 (PMC10919601; doi:10.1371/journal.pone.0298601)
Supplement: S1 Data — (ZIP) [file pone.0298601.s001.zip › Data/地级市Z_F工作报告.zip!/╔╜╬≈/╜·│╟╩╨/└┤╫╘╣1⁄2╓┌║┼í╛┬φ┐╦╩2╛▌═°í┐_2016.docx]

各位代表：

　　现在，我代表市人民政府向大会报告工作，请予审议，并请市政协委员和其他列席人员提出意见。

　　一、过去五年政府工作回顾

　　“十二五”时期是我市发展不平凡的五年。面对复杂严峻的宏观环境和艰巨繁重的改革发展稳定任务，在中共晋城市委的领导下，全市上下贯彻落实党中央、国务院和省委、省政府的各项决策部署，围绕“一争三快两率先”发展战略，积极适应经济发展新常态，扎实工作，努力作为，全市经济社会发展稳中有进、稳中向好。“十二五”各项目标基本实现。

　　五年来，我们多措并举稳增长，全市综合实力稳步提升。全市地区生产总值由2010年的730.5亿元增加到1040.2亿元，年均增长8.4%。全社会固定资产投资由2010 年的432.6亿元增加到1105.1亿元，年均增长20.6%。一般公共预算收入由2010年的55.5亿元增加到93.9亿元，年均增长11.1%。社会消费品零售总额由2010年的191.1亿元增加到358.8亿元，年均增长12.9%。外贸进出口总额由2010年的5.3亿美元增加到9亿美元，年均增长11%。2015年，城镇、农村居民人均可支配收入分别达到26651元、10914元，年均分别增长10.5%、12.8%。省、市两级重点工程累计完成投资3413.6亿元。

　　五年来，我们坚定不移促转型，产业结构不断优化。三次产业比重由2010年的4.2:63.6:32.2调整为2015年的4.7:55.4:39.9。粮食生产“五连丰”，现代特色农业势头良好。农业产业化经营组织达到225家，专业合作社5226家，农产品加工销售收入年均增长10.1%，农业机械化率达到70%。统筹做好煤与非煤两篇文章，煤炭资源整合和煤矿兼并重组基本完成，地方煤矿数量压减到94座，总产能达到9455万吨，以煤为基多元发展的煤炭产业新体系正在形成。坚持循环发展，两化融合，集聚集约，努力打造煤化工、电力、冶铸、陶瓷等传统产业升级版。装备制造、煤层气、现代煤化工、新材料、电子信息等新兴产业势头强劲，非煤工业增加值占规模工业以上比重达到42.1%，较“十一五”末提高23.7个百分点。金融保险、电子商务、商贸物流、文化旅游等现代服务业蓬勃发展，服务业增加值由235.2亿元增加到415.3亿元，年均增长7.6%。

　　五年来，我们坚持改革开放和创新驱动，经济发展内生动力和市场活力明显增强。认真实施转型综改三年实施方案和年度行动计划，“1+9”综改试点、低碳城市、智慧城市、气化晋城等稳步推进。简政放权、不动产登记、公共资源交易、商事制度改革等全面实施。积极开展“过桥还贷”、中小微企业贷款保证保险、产业投资基金、PPP等各类金融创新和农商行改制工作，多策并用帮扶实体经济。加强产学研合作，五年累计建成国家、省级企业技术中心14家、工程技术中心4家，国家高新技术企业达到34家。积极引导中小企业和民营经济发展，各类民营经济组织达到67865个，从业人员60.53万人。五年累计招商引资落地项目362个，资金到位2485.3亿元，实际利用外资13亿美元。晋城海关、商检、兰花保税物流中心正式运行，晋城经济开发区升级为国家级经济技术开发区。

　　五年来，我们坚持生态文明和环境保护并重，美丽晋城建设步伐加快。狠抓大气污染防治，突出“控煤、治污、管车、降尘”四个关键环节，“十二五”安排实施的202个重点节能减排项目全部完成，主要污染物排放量低于省控目标。全市公交车和出租车清洁能源应用率达到100%，主城区气化普及率达到95%以上，县城气化普及率达到90%左右。五年完成国省营造林面积达49.78万亩。新建绿道74公里，新建公园13339亩，新建湿地公园1300亩。中心城区环城生态圈建设正式启动。“井”字型大水网建设有序推进，《沁（丹）河流域生态修复和保护规划》、“三轴三线十一面”的《中心城区水系治理规划》编制完成。沁河继续保持全省最好水质，全市集中式饮用水源地水质100%达标。“十二五”期间，共创建国家级生态乡镇4个、省级生态乡镇15个和省级生态村42个。先后荣获“国家卫生城市”、“国际花园城市”、“国家森林城市”、“国家循环经济示范城市”等称号。

　　五年来，我们加强基础设施和公共服务设施建设，城乡面貌持续改善。全市人民期盼已久的太焦高铁开工建设。高平-沁水高速公路基本建成，阳城-济源高速公路已经开工，全市高速公路通车里程达到319公里。新建改建农村公路1040公里。中心城区五年累计投资162亿元，启动实施了64项城市道路工程，41条道路建成通车。金村低碳示范区、西北片区改造步伐加快。海会500千伏变电站建成投运，新增110千伏及以上输电线路481.72公里、变电容量496万千伏安，较“十一五”末分别增长了37.75%和102.8%。五年累计投入126.43亿元，开工建设各类保障性住房63217套，基本建成42808套，任务完成率135.5%。新增供水管网435公里、供热能力1500万平方米，城市（含县城）生活垃圾无害化处理率和污水处理率分别达到98%和94%，市区煤层气管网实现全覆盖。新增公交车辆521台，建成城市公共自行车系统。市级应急水源工程、重点引调水管道工程全部完工并投入运行，完成水保治理面积70.25万亩，32.37万农村人口的饮水安全问题得到解决，农村自来水普及率提高到95%。扎实推进大县城、特色镇、美丽乡村、产业片区建设和农村人居环境改善，城镇化率达到57.42%，年均提高1.3个百分点。完成采煤沉陷区治理搬迁15808户，易地扶贫搬迁19100人，农村危房改造7750户。巴公镇、北留镇、润城镇被命名为“国家级园林城镇”。

　　五年来，我们加大民生投入，各项民生事业和社会保障位居全省前列。2015年全市各项民生事业财政支出155.1亿元，占一般公共预算支出的84.5%，是2010年2.33倍。五年累计支出公共财政教育经费155.4亿元，新改扩建各类学校、幼儿园 273所。城区、泽州、高平、阳城通过国家义务教育发展基本均衡督导评估验收。基础教育成绩再创新高，高等教育实现历史性突破，太原科技大学晋城校区学生规模已达4000多名。“十二五”期间，累计投入18亿元，完成了晋城大医院门诊楼和6个县医院、77个乡镇卫生院（社区卫生服务中心）基础设施建设，村卫生室全部建成，村医补助标准提高到每月800元。市人民医院、晋城大医院、市妇幼院成功晋级三甲医院。市社会福利院迁建工程投入使用。积极开展分级诊疗和基本药物制度，实现了基本医疗保障和大病保险全覆盖。图书馆、档案馆、美术馆、博物馆相继建成开放，村级文化体育活动场所实现了全覆盖。五年累计新增就业18.83万人，转移农村劳动力13.23万人，完成新型职业农民培训1.8万人，城镇登记失业率控制在1.48%以内。建立创业基地24家，培养技能人才7.77万人、创业培训2.44万人，被国务院表彰为“全国创业先进城市”。全力实施精准扶贫，贫困村由496个减为200个，贫困人口由12.6万人减为8.45万人。社会保障水平逐年提高，城镇职工基本养老、城镇基本医疗、失业、工伤、生育、城乡居民养老保险人数较2010年增加120万人次，累计发放社会保障卡168.4万张，企业退休人员养老金待遇不断提高。“党政同责、一岗双责、齐抓共管”的安全生产责任体系全面建立，事故起数、死亡人数连续五年实现双下降。国防动员深入开展，连续三届荣膺“全国双拥模范城市”。妇女儿童、民族宗教、外事侨务、档案史志、残疾人、统计、气象、人防、地震、红十字、慈善、老龄等各项事业和援疆工作取得了新的成绩。

　　五年来，我们致力于民主法治建设和社会治理创新，政府自身建设不断加强。加强精神文明和民主法治建设，认真培育和践行社会主义核心价值观。自觉接受人大法律监督和工作监督，主动接受政协民主监督和社会监督。五年共办理人大代表建议1663件、政协提案1802件。“六五”普法工作全面完成。人民群众安全感和满意度测评连续四年取得全省第一，再次荣膺全国社会治安综合治理最高奖“长安杯”。严控机构编制和财政供养人员，压减“三公”经费支出，推进公务用车改革。完善依法科学民主行政决策机制，加快政府职能转变，新一轮政府机构改革全面完成。市县两级政府部门权力清单、责任清单全部按期公布。十八大以来，落实承接省政府取消、下放和调整的行政审批项目等事项426项，清理规范行政审批中介服务事项37项，取消市级行政审批事项70项，取消职业资格许可和认定事项63项。市级政务服务中心、公共资源交易平台、不动产登记交易中心基本建成。扎实开展党的群众路线教育实践活动、“三严三实”专题教育和“学习讨论落实”活动，严格执行中央“八项规定”精神和国务院“约法三章”，认真落实党风廉政建设“两个责任”，坚决反对“四风”，深入开展“三项治理”，查处和通报了一批违法违纪案件，弘扬了正气，凝聚了人心，为全市经济社会发展营造了良好的环境。

　　各位代表，过去的五年，我们通过艰辛的努力，取得这样的成绩来之不易。这是省委、省政府和市委正确领导的结果，是市人大、市政协有效监督、大力支持的结果，是全市人民齐心协力、共同奋斗的结果。在此，我代表市人民政府，向全市人民，向驻市解放军指战员、武警官兵和公安干警，向各民主党派、各人民团体和各界人士，向各位老领导、老同志，向所有关心、支持晋城发展的同志们、朋友们，致以衷心的感谢和崇高的敬意！

　　五年的探索实践，为“十三五”发展积累了宝贵的经验。这就是：必须坚持五大发展理念，自觉践行，贯穿始终；必须坚持以经济建设为中心，把全市干部群众的精气神和正能量凝聚到发展第一要务上来；必须坚持转型发展战略定力，不管形势如何复杂，无论遇到多大困难，都要把下决心转型的状态调整到最佳，把劲头铆到最足，不遗余力，久久为功；必须坚持改革开放创新驱动，激发经济内生动力和市场活力，更加注重发展的质量和效益；必须坚持统筹兼顾，“五位一体”协调发展；必须坚持以人民为中心，让改革发展成果更好地惠及广大人民群众；必须坚持抓铁有痕、踏石留印的实干精神，持之以恒、一抓到底，干在实处、争创一流。

　　五年来，虽然我们取得了不少成绩，但也必须清醒地看到，当前仍是我市建市三十多年来经济社会发展最为艰难的时期。发展不平衡、不协调、不可持续的问题依然突出：一是以煤为主的产业结构单一，非煤产业发展慢、比重小的问题突出，这一短板一直没有突破性改观；二是开放意识不足，体制机制改革创新滞后，经济增长内生动力和活力不足；三是经济社会粗放式发展与日趋严格的能源消费管理及资源环境的矛盾更加突出；四是“三期叠加”和产能过剩引发的各种社会风险逐步凸显，部分企业生产经营困难；五是一些干部担当不够的问题比较突出，难以适应新常态要求。

　　面对前所未有的困难局面，我们必须把思想和行动统一到省委“一个指引、两手硬”的重大思路和要求上来，统一到市第七次党代会精神上来，不断强化发展的战略思维、创新思维、法治思维、市场化思维和互联网思维，持续推进“一争三快两率先”战略步伐，为建设经济强市、美丽晋城而努力奋斗。

　　二、“十三五”时期政府工作的主要目标和任务

　　“十三五”时期，是全面建成小康社会的决胜期，也是重铸晋城辉煌，实现振兴崛起的关键期。我们能否建成小康社会，率先走出一条资源型地区创新驱动转型升级的新路，实现凤凰涅槃，浴火重生，关键看我们贯彻中央、省、市决策部署的执行力，关键看我们干部群众干事创业的激情和活力。面对历史赋予的千钧重担，面对多年来少有的困境时艰，面对市场倒逼转型的重大机遇，我们必须凝心聚力，众志成城，必须夙夜在公，只争朝夕，主动作为，以非凡的责任和担当，以超常的干劲和闯劲，以强烈的使命感和紧迫感， 奋力开创经济社会发展的新局面！

　　综合分析当前和今后发展面临的形势，结合国际、国内发展大势和我市实际，今后五年政府工作的指导思想是：高举中国特色社会主义伟大旗帜，深入贯彻落实习近平总书记系列重要讲话精神，按照“五位一体”总体布局和“四个全面”战略布局，围绕省委“一个指引、两手硬”的重大思路和要求，认真落实市第七次党代会精神，深入推进“一争三快两率先”战略部署，确立“三大”发展思路，实施“十大战略工程”，把晋城打造成展示“中国梦”、实现“两个百年”目标的样板市。

　　今后五年我市经济社会发展的主要预期目标是：地区生产总值年均增长6.5%左右，服务业比重年均提高1个百分点以上，研究与试验发展经费投入强度达到2.5%，常住人口城镇化率达到60%以上，户籍人口城镇化率达到44%，森林覆盖率达到44.5%，节能减排等约束性指标控制在省下达指标以内，实现综合经济实力、创新创业活力、社会文明程度、人民生活品质、生态环境质量“五个明显提升”。

　　今后五年政府工作确立的“三大”发展思路是：要把大开放、大转型、大创新，始终作为遵循和把握实现“五个明显提升”的立足点和基本点，为促进经济社会发展打好基础，做好保障。

　　要全面实施大开放。开放的不足，是晋城发展最大的不足。与先进发达地区比，我市的差距在开放、潜力在开放、希望在开放。我们要始终把大开放作为牵动全局的关键举措，跳出晋城看晋城，立足全球看晋城，面向未来看晋城，主动对接国家战略，充分利用两个市场、两种资源，加快各类开放平台的建设，全面拓展开放发展的新空间。特别是要主动承接东南沿海产业梯度转移，以积极有为的态度投身国家“一带一路”发展战略，努力形成思想观念的大开放、行为行动的大开放、服务水平的大开放、投资环境的大开放格局。

　　要全面实施大转型。转型发展迫在眉睫，跨越发展时不我待。大转型是晋城发展的唯一出路。我们要把“大转型”作为树立晋城美好形象、实现晋城振兴崛起的重大举措，坚决丢掉“再现煤炭十年黄金期”的幻想，坚决克服“不想转”的思想和“不会转”的困局。“十三五”期间，市政府将设立转型发展资金，加快推动大转型的落地成型，真正把转型变成项目，把转型变成企业，把转型变成市场。以重大项目推进和领军企业为抓手，着力打造一批转型的好项目大项目，着力建设一批具有竞争力的产业集群，着力发展一批具有引领作用的龙头企业，全面推进传统产业的大转型，全面推进新兴产业的大发展，全面推动全市工业整体素质和竞争力的大提升，真正走出一条具有晋城特色的转型升级发展新路。

　　要全面推进大创新。创新是引领发展的第一“引擎”。我们要把创新摆在发展全局的核心位置，把大创新当作硬任务，以创新考绩，以创新论功，着力激发大创新的内生动力和强大活力。要突出抓好大创新的“顶层设计”，加快制定晋城创新驱动发展的战略规划，加快形成大创新发展的新机制，着力打通创新向现实生产力转化的通道，让创新成为增长点，让创新成为增长极，让创新成为增长带。要鼓励创新，要宽容失败，把人才作为创新的第一资源，把奖励优秀放在突出的位置。要营造出一种创新无处不在，创新贯穿一切的大创新环境。

　　按照以上指导思想、发展思路和目标，“十三五”期间，要重点实施“十大战略工程”，全面提升市域发展综合实力。

　　1、转型突破工程

　　未来五年，我们要确立100项工业转型项目，加快推进五大产业的转型。一是煤炭产业，要坚持以煤为基，“立足煤、延伸煤、超越煤”，做好煤与非煤两篇文章。要保持煤炭这个基本面，紧紧盯住清洁高效利用这个终极目标，加快推进煤炭由燃料向原料、由低品位向高附加值、高端化的转型。加快推进煤炭材料化和煤基新材料技术的突破，全力攻关煤基领域科技创新链和高新技术领域创新链延伸，晋煤、兰花、晋能等大型煤企要积极带头落地一批转型项目。通过坚决转、大幅转、创新转，把晋城打造成高碳资源低碳发展、黑色资源绿色发展的煤炭高科技先行区。二是煤化工产业，要瞄准国际标准、行业标准，牢固树立“高科技+现代资本＝先进生产力”的理念，构建“集成联产、深度加工”的产业发展新体系，加快谋划一批现代化高端新型煤化工产业项目。重点发展煤基新材料和煤基新能源，以高端化技术培育高端化产业、产品，把进入产业的各种资源“吃干榨尽”，实现循环、环保、节能的产业发展目标，带动煤基材料向精细化产品、向航天航空等高科技领域产品转型升级。晋煤华昱、天泽、兰花、晋能等大型企业要坚持以高科技、多联产、深加工为方向，大力延伸煤化工产业链条，壮大现代煤化工产业集群，形成支撑现代煤化工产业发展的高端支柱产业，大幅度提升高硫煤洁净利用化工产业的竞争优势。力争到“十三五”末，全市煤化工年销售达到500亿元，利税100亿元，把我市建成国内一流的现代煤化工高科技产业城。三是煤层气产业，要加快智能化大数据信息的运用，引领煤层气产业向国际化、高端化发展。要加快实现煤层气+互联网+物联网+云计算的智能化新载体效应，加快发挥大数据平台对全产业链数据的高效传输共享和全面提升产业科技成果高效利用的能力。同时，煤层气企业要利用上游资源的优势，延伸中下游产业，要以气源的开采规模带动形成一批高附加值产品产业的发展。要坚决克服“只抽、只卖、不用”的现状，大幅提高就地转化的能力，充分把握资源市场的主动权，改变资源大市与产业话语权不匹配的现状，变被动的买方市场为主动的卖方市场。把煤层气产业打造成全国一流的煤层气开发利用及就地转化高附加值产品的产业基地，实现“气”资源和“气”商品的双赢。四是煤机装备制造业，依托金鼎煤机、江淮重工、天巨重工等企业，全面打造“晋城煤机”品牌，加快煤机制造的资源整合，加快煤炭采掘、支护、运输、钻探、洗选成套煤机装备的制造，建设全省生产能力最大、生产技术最优、服务煤矿能力最强的晋城煤机产业集群。加快把金匠园区建成晋城煤机高新项目的孵化中心、技术工艺创新中心、煤机装备研发中心、高端发展信息中心。加速淘汰落后采煤工艺及装备，促进晋城自主知识产权的创新型采煤工艺和装备，提高扩大市场的占有能力和占有率。五是煤电气一体化发展。全面推进煤电一体化、采煤采气一体化深度融合发展。推进电力企业和煤炭企业实施联营合作，优化电力产业布局，形成以超临界燃煤机组为主的全球规模最大的火电产业集群和以煤层气利用为主的清洁能源发电产业集群。要以晋东南大型煤电基地为重点，发展大容量、高参数超临界、超超临界燃煤机组，大力发展煤层气发电与煤矸石、煤泥等低热值燃料发电。大力推动外送电通道建设，扩大直供电领域和规模。阳电三期、国投二期、高平电厂要积极争取上马建设，要确保农网改造升级工程的实施，要重点抓好泽州华电、沁水远景汇合、陵川中电投三个风电项目的建设。推进电力基地与电网建设一体化的发展。

　　2、增长培育工程

　　增长培育工程旨在培育壮大新兴产业，是促进经济增长方式转变的一条捷径，是衡量新兴产业培育水平的标志。“十三五”时期，要重点加快光机电、高端智能化装备制造、新能源新材料、生物医药、现代服务业五大新兴产业的发展。一是大力推进光机电产业。发展光机电产业是晋城工业走向高科技领域的标志。我们要从关乎我市工业结构调整的高度，举全市之力把富士康做大做强。千方百计把富士康在海外核心技术、核心产业转移到晋城，下大决心支持富士康把晋城建成世界一流的光纤产品基地和世界重要的光机电产业基地，打造中国北方“光谷”。我们要珍惜郭台铭先生坚守的“在家乡投资，只能成功、不能失败”的决心，这份坚守，是一位游子回报家乡的真情，我们要感激老乡。在此，请允许我提议，对郭台铭先生对家乡的关心、支持再一次表示衷心的感谢！更希望富士康在现有光通讯连接器、光学镜头模组、精密刀具、手机构件等产品基础上，把手机整机生产线、白色家电项目、液晶玻璃等高端产品投在晋城，努力实现五年“再造一个富士康”。力争到“十三五”末，全市电子信息产业年销售收入达200亿元，利润50亿元，把晋城建成世界一流的光机电高科技产业城。二是大力推进高端智能化装备制造产业。重点抓好先进智能装备制造，加快节能环保、基础工艺装备发展。依托江淮重工等龙头企业，重点发展工业机器人、核聚阀门、风能等高端智能装备。依托天巨重工、天泽太行机械等骨干企业，发展 30 万千瓦以上火电机组及钢厂等脱硫脱硝和袋式除尘技术成套设备，及资源再利用成套设备。坚决支持中船重工江淮集团新能源装备产业园区的发展，加快风电总装基地的建成。同时，要加快整合铸造行业生产要素，引导全市百家铸造企业向园区集中。通过集中提升铸造业的科技含量和科技水平，推动铸造业由“晋城铸造”向“晋城制造”和“晋城智造”的根本转变。三是积极发展新能源新材料产业。新能源产业，要加快培育分布式太阳能光伏发电、太阳能光热资源利用。重点围绕电动汽车整车制造、关键零部件、关键装备、充电配套设备等方面，培育锂离子动力电池、光伏电池组件产业的发展，力争五年内，重点打造3-5家电动汽车及关键零部件骨干企业，加快把晋城建成中西部地区和全省重要的电动汽车生产基地。新材料方面，重点发展化工新材料、高性能结构新材料、陶瓷新材料和前沿新材料。积极推进适合山西高灰熔点及低质煤大规模粉煤气化的技术，积极推进“晋煤炉”、“航天炉”无烟煤三高劣质煤技术的攻关，有序发展煤制甲醇、煤制芳烃等化工新材料，发展己内酰胺、三聚氰胺等精细化工产品和烯烃衍生产品。依托路宝、霖春镁业、蔡欣金属钙镁等企业，重点发展铝镁合金压铸件及其深加工产品，加快培育我市铝镁合金铸件产业发展。以阳城陶瓷为依托，积极承接国内陶瓷基地产业转移，重点开发陶瓷干法技术、低温快烧、高强度薄砖等技术，发展多孔陶瓷和蜂窝陶瓷。同时，要积极培育富基新材料、海诺科技高性能空心玻璃微珠、兰花华明纳米新材料、天坤特种刚玉纳米新材料等龙头企业发展。四是推动生物医药产业快速发展。发挥本地党参、柴胡、连翘等中药材资源优势，建设符合国家GAP要求的中药材种植基地，推进中药材种植、加工、生产、销售的产业化。重点发展中药材深加工产业集群和中成药产业集群。在中药材示范基地基础上，进一步优化药材品种，扩大生产规模，提高产品质量。依托中成药生产优势，大力进行中成药创新研发，通过合作开发、项目引进等手段，力争在心血管、高血压、儿童医药等领域实现突破，形成以中药针剂、中药饮片、中药养生保健品等技术为主的中药提取和合成中心。五是加快发展现代服务业。主动适应生活性服务业转型升级、生产性服务业加快发展的现实态势和客观需求，以文化旅游为龙头带动，大力发展现代物流、信息技术、节能环保、电子商务、文化创意、健康养老等现代服务业，推动向精细化、规模化和高品质方向转变，构建“高增值、强辐射、广就业”的现代服务业体系。特别要大力发展金融业，发展普惠金融和绿色金融，实施企业上市培育行动，成立担保集团，设立产业投资基金和股权投资基金等，强化金融供给效益，构建体系健全、竞争有序、运行规范、监管科学、与实体经济发展相适应的现代金融服务体系。力争到2020年，服务业增加值占GDP比重达到45%以上。通过加快五大新兴产业发展，到“十三五”末，培育出超百亿增长的新的增长极，为全市发展增加新的动力。

　　3、城市提质工程

　　城市提质工程是引领全市区域综合经济发展的一个载体，是争先综改、竞逐中原的资本和平台。“十三五”期间，要坚持以中心城区为龙头，优化中心城市功能为核心，依托“拓展、改造、提质”总要求，进行科学区划，全力打造区域中心城市。启动城市总规修编，拉大城区基础框架。打造以主城区为中心，以晋煤新区、富士康新区、金村新区、高铁新区、空港新区为框架的六区联动、组团发展的中心城市框架。重点实施中心城区提质的六大工程，实施主城区与各县（市、区）、主城区及各片区与高铁站快速干线连接工程，与机场快速通道、城市内环、城市外环十条环城快速通道的连接工程；实施中心城区百条街巷道路畅通工程，打通城市“断头路”，启动城市互通式立交桥建设，畅通城市主街道，打通城市微循环；实施煤层气地下管廊全覆盖工程，建设“双气源”供气保障体系，保障全市人民用上清洁高效能源。启动“大热源”工程，加快阳电供市区供热工程的建设。启动“大水源”工程，加快建设以张峰水库供水工程为主的十大蓄水工程、三处引水和四处提水工程，年增加供水能力1.04亿立方米，保证全市人民喝上干净的水，确保饮用水的安全。加快建设一批停车场、立体车库、菜市场、早夜市摊点、公厕等便民服务场所，优先发展公共交通，有效缓解“城市病”。以建设国家智慧城市为契机，综合运用物联网、云计算、大数据等现代信息技术，推进城市管理和城市服务“双提升”。同时，要牢固树立“发展区域经济、彰显文化灵魂”的理念，向城市注入大量的地域文化元素，把晋城悠久的历史文化重新写进城市。到“十三五”末，努力把中心城区建成百平方公里、百万人口“双百”规模的大市区，把大市区打造成吸引中原财富智慧的聚集地。

　　4、招商引智工程

　　要全面实施大招商和大引智。要用系统和定向的观点改进招商引资工作，下大气力研究市场需求，下大气力谋划项目。要用我们的资源优势、产业优势、市场优势、利润空间确定招商方向，构建大招商、招大商、大投资、大效益的招商引资双赢模式。实现招商引资项目的大落地，实现招商引资总量的大突破，让晋城成为海内外投资兴业的热土。同时，要充分认识头脑风暴、专家智囊在经济发展中的重要性，加快百名院士博士工作站的建设，提升晋城科研综合实力。“十三五”期间，我们要加大各类高层次人才的引进力度，通过办好一个“文化节”、筹备好两个“博览会”、主办好三个“国际论坛”，来搭建招商引智的平台。要紧紧抓住举办“中国晋城太行山旅游文化节”的契机，展示晋城深厚文化底蕴，提升我市在国内外的知名度和影响力。要着眼于我市装备制造业以及特色农业产业良好的发展基础和潜力，筹办“中国晋城装备制造业博览会”和“中国晋城特色农产品博览会”，集中展示装备制造业以及特色农业的发展的水平，通过举办“展会”，搭建产学研合作的平台。要积极谋划在我市举办国际煤层气晋城论坛、全国装备制造业晋城论坛和国际新能源新材料晋城论坛，通过举办论坛，搭建院士博士晋城工作站实验室工作平台。力争到“十三五”末，高标准建成国际级煤层气专家工作站、国际级光机电专家工作站、国家级现代煤化工专家工作站、国家级新能源新材料专家工作站、国家级铸造装备专家工作站、国家级市域经济发展规划专家工作站、国家级城市建设规划专家工作站、国家级生态经济发展专家工作站、国家级旅游经济发展专家工作站、国家级金融创新发展专家工作站等10大工作站，促使高新技术企业成规模增长，促使科技进步对经济增长的贡献率快速提升。使晋城成为高端人才、院士科学家聚集的福地、高新技术发明的产地、科研成果转化的基地，进入创新城市和人才强市的行列。

　　5、通道快捷工程

　　交通是发展的命脉，道路是开放的出口，大通道是经济社会发展的“大纽带”，是人流、物流、信息流的“大走廊”。“出不去，进不来”是我们晋城发展最大的“瓶颈”。我们要把大通道建设作为服务经济发展和改善民生的重要任务，建设大通道、培育大枢纽。铁路建设，确保“十三五”内太焦高铁建成通车，实现晋城高铁“零”的突破；续建嘉峰至南陈铺的普通铁路，形成侯月铁路与太焦铁路的连接线。机场建设，通用机场力争“十三五”末建成并投入使用，实现晋城机场“零”的突破；民用机场力争开工建设。高速公路，以晋城现有高速公路为基础，打通陵侯高速向东至鹤壁，贯通晋城高速公路东出口；续建高平至沁水高速公路，建设阳城至蟒河、安泽至沁水高速公路，形成晋城“两环两横三纵十出口”的高速公路大格局。普通公路，加快普通国道、省道、干线公路改造升级，实现县（市）现有国道达二级以上，省道达三级以上，县与县通一级公路。加快农村公路建设，加大交通扶贫力度，实现农村公路“建好、管好、护好、运营好”四好公路目标。力争到“十三五”末，把晋城交通建设成为“连接东西、沟通南北，大出大进、快出快进，立体式全面开放”的大通道。

　　6、园区建设工程

　　园区建设既是投资环境，也是发展环境。家有梧桐树，才有凤凰来。“十三五”时期，我们将以“一区九园”为重点，推动园区建设上规模、上档次、上水平，打造产业特色明显、竞争优势突出、平台功能完善的工业园区。整合全市各类产业园区，市级重点建设晋城经济技术开发区，各县（市、区）要集中力量打造一个工业园区。力争“十三五”时期，各县（市、区）要建成一个省级以上开发区。按照“管委会+公司”运作模式，组建园区开发建设公司，优化政府和市场两种资源配套的能力和手段，加快园区开发建设。高质量建设园区基础设施，实现水、电、路、气、供热、排水、排污、治污及网络等基础设施配套完善。加强园区生产、生活、政务、商务等公用配套设施建设，全面提高园区配套服务水平。积极争取先进地区和开发区在我市建设飞地产业园，实行跨区域异地托管、开发、收益分成共享。创新园区管理体制和内部机制，推动园区全力抓招商、全力抓项目，把园区建设成为招商引资、产业集聚的桥头堡和示范区，引领全市经济转型升级。力争到“十三五”末，全市工业园区总面积达到100平方公里，总产值超1000亿元。

　　7、碧水蓝天工程

　　生态是晋城最大的资源优势，生态建设是晋城永恒的主题。我们要通过大力实施碧水蓝天工程，开展生态修复和环境治理，让绿色染遍“太行屋脊”，让生态扮靓“天下公园”。要继续加大环城生态圈的建设。持续开展绿化提升行动，初步形成八大生态片区396平方公里的环城生态圈，建成环城绿道270公里，新建城市公园5个和街头绿地35处，继续实施重点林业生态工程建设，加快三山、四片、五环、20条线、70个乡镇、300个村绿化工程，筑牢全市域绿色生态大屏障。着力推进国家低碳城市试点和循环经济示范市建设，推广绿色建筑，发展绿色交通，营造绿色消费环境，倡导绿色低碳生活。要加快实施沁丹两河“清水复流贯通工程”。以沁河、丹河流域水生态修复与保护为重点，以“清淤、活水、保洁、生态”为主题，建设以张峰水库供水工程为龙头、覆盖全市城乡的两纵两横“井”字型大水源工程。抓紧推进沁河、丹河、长河、固县河等主要河流沿河小型水利灌溉工程建设。加强城镇污水处理设施配套工程，对城区西河、东河等4条河流和沁河、丹河城区段进行综合治理，实现雨污全分流、管网全封闭、清水活流全贯通。要全面加强环境综合治理。深入实施大气、水、土壤污染防治行动计划，抓好主要污染物减排工作，积极发展清洁能源，推动重点行业能效提升和节能减排升级改造。到“十三五”末，实现城市主干河道清水复流全贯通，让清水、活水真正用起来、环起来、流起来，让我们的城市因水而秀美、因水而灵动、因水而和谐，让美丽晋城山连山、水连水、山更青、水更绿、天更蓝、城乡更美丽、生态更美好，实现“城在林中、林在城中、人在绿中”的天下公园大景观。

　　8、全域旅游工程

　　旅游业是新常态下新的经济增长点。“十三五”时期，我们要叫响美丽晋城全域游品牌，积极申报“国家全域旅游示范市”，打造全域旅游产业。编制《美丽晋城战略规划工程》纲要和《晋城市全域旅游发展五年规划》，改革创新旅游综合管理体制、市场监管机制，完善旅游公共服务，提升旅游业发展综合能力。促进旅游业与休闲农业、特色工业、健康养生、文化创意、传媒娱乐等业态融合发展，形成共建共享美好生活、共建共享基础设施、共建共享公共服务、共建共享生态环境的旅游发展大格局。加快特色小镇建设，实施乡村旅游富民工程。以沁丹两河流域为重点，突出特色文化传承，重点建设一批以司徒、大阳为代表的特色商贸重镇，以润城、北留为代表的历史文化名镇，支持阳城、陵川、沁水建设一批历史文化名人和生态旅游大镇。到“十三五”末，要高标准建设30个产业特色鲜明、人文气息浓厚、生态环境优美、多功能叠加融合、体制机制灵活的特色小镇，建成100个美丽宜居示范村。要加强旅游基础设施建设，加强旅游市场监管，加强智慧旅游建设，增强综合服务能力。推广精品旅游线路，深度挖掘晋城历史文化、自然生态、红色文化等资源，发展特色旅游产品，着力打造沁河古堡休闲探秘、太行山水、析城山——历山自然保护区、炎帝文化寻根问祖4条精品旅游线路。加强旅游促销，发挥“晋善晋美晋城”品牌效应，开通皇城旅游专列，开通太行山、析城山、历山旅游快车，开通炎帝寻根快线，为全域旅游发展聚集人气。努力把文化旅游业培育成我市的战略性支柱产业，到“十三五”末，在全域旅游发展方面走在全省前列。

　　9、精准脱贫工程

　　精准脱贫工程是全面建成小康社会的攻坚工程，是一项政治任务，是一项民心工程。要坚持把脱贫攻坚作为“十三五”期间的头等大事来抓，用更大的决心、更准确的思路、更精准的举措打赢这场“输不起的攻坚战”，确保在全面建成小康社会中“一个不掉队”、“一个不能少”。未来五年，要认真落实“六个精准”脱贫方略，确保建档立卡的贫困村和贫困人口较多的低收入村实现干部驻村帮扶工作队全覆盖，确保贫困户实现党员、干部结对帮扶全覆盖，确保沁水县、陵川县2017年脱贫摘帽，确保所有贫困村、贫困人口2018年底前全部脱贫；其他非贫困县贫困村、贫困人口2017年底全部脱贫。精心实施“五个一批”扶贫工程，完成易地扶贫搬迁23887人目标。加快发展现代农业，要把畜牧、蚕桑、食用菌、设施蔬菜、小杂粮、干水果、苗木花卉等优势特色农业，作为脱贫的重要产业来支持，积极发展城郊型创意高效农业、休闲农业、观光农业和农村电子商务等新业态发展，帮助3.3万人通过产业脱贫致富。继续提高农村低保标准，完善农村贫困人口医疗制度，加大医疗救助、临时救助、社会救助等帮扶力度，帮助完全或部分丧失劳动能力的3.45万贫困人口依靠社会保障兜底脱贫。用好中央、省里政策红利，创新PPP模式，引导社会资本投入贫困地区，实施“金融支持特色产业发展富民扶贫工程”，实现金融小额贷款扶贫全覆盖。“十三五”时期，要实现建档立卡确定的200个贫困村、8.45万贫困人口彻底全部脱贫。

　　10、民生福祉工程

　　改善民生是一切工作的出发点和落脚点，人民群众对美好生活的向往就是我们努力工作的目标。我们只有带着最深厚的感情去倾听群众呼声，了解群众疾苦，办好为民实事，才能让人民群众拥有更多的获得感、幸福感。未来五年，要在七个方面抓好大提升工程。一是教育大提升。进一步调整优化城乡中小学布局，市区新建一批中小学校。全面普及高中阶段教育，规范发展民办教育，提升发展特殊教育。特别要针对学前幼儿入园难、入园贵的问题，有序建设布局一批公办幼儿园，同时，通过政府购买服务的方式，为群众提供普惠性的学前教育服务，提高幼儿园的办学条件，满足社会的迫切需求。加快金村教育园区建设，组建职业教育集团，扩展太原科技大学晋城校区学科专业，更好服务于地方经济。二是卫生大提升。以解决看病难、看病贵为重点，深化医疗体制、计划生育服务管理“两大改革”，推动医疗、医保、医药三医联动，健全市县乡村分级诊疗服务网络，提高城乡居民医疗保险标准，补齐农村医疗卫生短板，优化中心城区医院布局。重点加快晋城大医院投入使用，加快市人民医院易址扩建，加快启动市中医院建设。在“十三五”末，使全市人民在医疗卫生方面得到一个大提升。三是文化大提升。继续实施文化惠民工程，广泛开展全民阅读、全民艺术普及、全民健身文化活动，推动公共文化服务标准化、均衡化发展。大力发展具有晋城传统文化特色的文化服务业，推出一批地方特色的精品佳作。完善公共文化基础设施，筹备启动市民需求的科技、文化、自然博物馆的建设工程。到“十三五”末，通过丰富地域文化，让晋城人民不出晋城就能享受到丰富的文化生活，使文化设施得到大改善、群众文化生活得到大提高。四是就业创业大提升。继续深化多渠道、多形式灵活的就业扶持政策，发展“众创、众包、众扶、众筹”新模式，健全劳动者自主择业、市场调节就业、政府促进就业相结合的体制机制。加强创业公共服务平台建设，建设改造一批示范性创业孵化基地和创业园区，提高国家级创业型城市建设水平。到“十三五”末，全市城镇新增就业达13万人，创业就业人数达4万人，转移农村劳动力达12万人，城镇登记失业率控制在4.5%以内。五是社会保障大提升。实施全民参保计划，全面建设和完善覆盖城乡的社会保障体系。推进机关事业单位养老保险和城镇住房公积金制度全覆盖，完善城乡居民基本养老保险、大病保险、最低生活保障和医疗救助制度。支持社会力量发展养老服务业，发展儿童福利事业，支持公益慈善事业发展。到“十三五”末,要建成更加完善的、覆盖全体城乡居民的社会保障体系，基本养老保险参保率达到90%，养老服务机构达到100所，让人民老有所养、病有所医。六是安全生产大提升。生命高于一切，安全重于泰山。要认真落实总体安全观，按照省委提出的“三个坚决防止”的要求，坚决守住安全稳定的底线。坚持“三个决不能过高估计”和“三个敬畏”，毫不懈怠地做好各行业、各领域安全生产工作。认真落实“党政同责、一岗双责、齐抓共管”安全生产责任制，坚持管行业必须管安全，管业务必须管安全，管生产经营必须管安全，夯实安全生产基层基础建设，健全安全生产管控体系。深入开展重点行业领域专项整治，保持全市安全生产形势平稳好转。七是社会治理大提升。创新社会治理模式，建立社区、社会工作者、社会组织“三社联动”机制。严厉打击各类违法犯罪活动，健全社会治安防控体系，完善信访工作和矛盾调处机制，维护社会和谐稳定。加强食品药品安全监管，保障饮食用药安全。加强应急管理，提升防灾减灾能力。加强国防动员，促进军民融合发展。此外，还要大力发展妇女儿童、老龄、慈善和红十字会等事业。做好气象、地震、科普、档案、史志、民族宗教、外事、侨务、对台、对口援疆等工作。

　　总之，通过实施十大战略工程，到“十三五”末，确保全市生产总值、城乡居民人均可支配收入比2010年翻一番，人均GDP、人均公共财政预算收入等主要指标排位，进入中原经济区5省30市的“第一方阵”。

　　各位代表，完成“十三五”目标任务，必须切实加强政府自身建设，创新管理方式，提升服务效能，努力打造法治政府、创新政府、廉洁政府和服务型政府。要全面推进依法行政。完善依法决策机制，实行规范性文件备案审查和法律顾问制度。加强行政复议和行政执法监督。深入开展“七五”普法工作。严格执行人大及其常委会的决议、决定，自觉接受人大监督、政协监督、社会监督和舆论监督，让行政权力在法治轨道和阳光下运行。要加快政府职能转变。深入推进放、管、服改革，加强事中事后监管。优化公共服务流程，市级政务服务中心投入运行，健全为企业、为群众、为基层服务的各项制度，守信践诺，说到做到，全面提升服务水平。要狠抓工作落实。大力弘扬晋城人不甘人后、勇争第一的精神和勇气，激发各级干部的担当使命，建立“马上就办、真抓实干”工作机制，最大限度调动各方面干事创业的积极性和创造力，把各项工作真正落到实处。要加强党风政风建设。坚持把纪律和规矩挺在前面，深入开展政府系统党风廉政建设和反腐败斗争。严格落实中央八项规定、国务院“约法三章”和省市各项规定，自觉践行“三严三实”，深入开展“两学一做”学习教育。强化行政监察和审计监督，加强对重点领域、关键环节、重要岗位权力运行的监督制约，确保各项政策的贯彻落实。

　　各位代表，2016年，是“十三五”规划的开局之年。在年初召开的市委六届六次全会暨全市经济工作会议上，对今年的工作已进行了安排部署，各项工作正在积极有序推进，经济社会发展正在奋力前行。现在离年底还有两个多月，各项工作时间紧、任务重。我们要按照省委“一个指引、两手硬”重大思路和要求，深入贯彻市第七次党代会精神，紧紧咬住既定目标不放松，大干70天，全力巩固经济低位企稳回升态势，力争“一月比一月好、下半年好于上半年”，为明年进一步好转奠定坚实基础。要把稳定增长作为重中之重，推进供给侧结构性改革，全力扭转工业下滑态势，确保今年扶持“1533工程”的九条政策落地兑现。要打好环境保护攻坚战，落实好今年确保完成目标任务、明后两年进入并稳定在全省前列、“十三五”末力争成为全省标杆的“三步走”计划，加大环境治理力度，持续改善人居环境。确保“十三五”各项工作开好局、起好步。

　　各位代表，实现“十三五”经济社会发展的宏伟蓝图，建设幸福美好现代化城市，责任重大、使命光荣。让我们紧密团结在以习近平同志为总书记的党中央周围，在省委、省政府和市委的坚强领导下，凝心聚力，砥砺奋进，深入推进“一争三快两率先”战略，为实现“十三五”目标，努力在全省率先走出资源型地区创新驱动转型升级发展的新路、率先全面建成小康社会而努力奋斗！
